# Early toxicity predicts long-term survival in high-grade glioma

**DOI:** 10.1038/bjc.2011.123

**Published:** 2011-04-12

**Authors:** Y R Lawrence, M Wang, A P Dicker, D Andrews, W J Curran, J M Michalski, L Souhami, W-Ka Yung, M Mehta

**Affiliations:** 1 Department of Radiation Oncology, Thomas Jefferson University, Kimmel Cancer Center, Bodine Cancer Center, 111 S. 11th Street, Philadelphia, PA 19107, USA; 2 Department of Radiation Oncology, Sheba Medical Center, Tel HaShomer, 52621, Israel; 3 Department of Statistics, Radiation Therapy Oncology Group, 1818 Market Street, Suite 1600, Philadelphia, PA 19103-3604, USA; 4 Department of Neurological Surgery, Thomas Jefferson University, 111 S. 11th Street, Philadelphia, PA 19107, USA; 5 Department of Radiation Oncology, Emory University School of Medicine, 1365 Clifton Road, Atlanta, GA 30322, USA; 6 Department of Radiation Oncology, Washington University School of Medicine, 660 South Euclid Avenue, Campus Box 8224, St Louis, MO 63110, USA; 7 Department of Radiation Oncology, McGill University, 1650 Cedar Avenue, Montreal, Quebec H3G 1A4, Canada; 8 Department of Neuro-Oncology, University of Texas, MD Anderson Cancer Center, 1515 Holcombe Boulevard., Houston, TX 77030, USA; 9 Department of Radiation Oncology, Northwestern University Feinberg School of Medicine, 251 East Huron Street, Chicago, IL 60611, USA

**Keywords:** glioblastoma, toxicity, normal tissue effects, radiation therapy

## Abstract

**Background:**

Patients with high-grade gliomas are treated with surgery followed by chemoradiation. The risk factors and implications of neurological side effects are not known.

**Methods:**

Acute and late ⩾ grade 3 neurological toxicities (NTs) were analysed among 2761 patients from 14 RTOG trials accrued from 1983 to 2003. The association between acute and late toxicity was analysed using a stepwise logistic regression model. The association between the occurrence of acute NT and survival was analysed as an independent variable.

**Results:**

There were 2610 analysable patients (86% glioblastoma, 10% anaplastic astrocytoma). All received a systemic agent during radiation (83% chemotherapy, 17% biological agents). Median radiation dose was 60 Gy. There were 182 acute and 83 late NT events. On univariate analysis, older age, poor performance status, aggressive surgery, pre-existing neurological dysfunction, poor mental status and twice-daily radiation were associated with increased acute NT. In a stepwise logistic regression model the occurrence of acute NT was significantly associated with late NT (OR=2.40; 95% CI=1.2–4.8; *P*=0.014). The occurrence of acute NT predicted poorer overall survival, independent of recursive partitioning analysis class (median 7.8 *vs* 11.8 months).

**Interpretation:**

Acute NT is significantly associated with both late NT and overall survival.

Second to meningioma, high-grade gliomas (WHO grade 3, 4) are the most frequent type of primary brain tumours in adults. Treatment consists of maximal safe resection followed by partial brain radiation. Following the introduction of concomitant and adjuvant temozolomide, long-term survival for grade 4 gliomas (glioblastoma, GBM) has improved, with almost 10% of subjects now living 5 years (Stupp *et al*, 2009). The long-term toxicity of treatment is, therefore, of increasing importance.

Patients with high-grade glioma undergoing chemoradiation experience various side effects, including dermatological, endocrine, systemic and neurological events. Dermatological side effects such as radiation dermatitis and alopecia occur early and are generally transient, although alopecia may take several months to reverse. These rarely interfere with functional independence, but may contribute significantly to a reduction in quality of life because of a diminution in self-worth. Endocrine side effects are usually delayed by several months to years, are gradual in onset and often subtle, in terms of clinical presentation, and hence are underdiagnosed; they are more frequent in children than adults (Cross and Glantz, 2003). Systemic side effects such as myelosuppression and diarrhoea are generally attributable to chemotherapy.

Neurological side effects occur both early and late. Acute effects (within 90 days of the commencement of therapy) are often transient and include fatigue, headache, nausea, motor/sensory disturbances, raised intracranial pressure, cranial nerve palsies, visual disturbances, seizures and subtle changes in short-term memory. Late side effects (more than 90 days after the commencement of therapy) include many of the same symptoms, with the addition of cognitive decline (Taphoorn and Klein, 2004), cerebellar dysfunction and the consequences of white matter atrophy such as normal pressure hydrocephalus; these are rarely reversible.

It is often impossible to determine whether such neurological symptoms are side effects of radiation therapy (RT), surgery, chemotherapy, medications (e.g., anti-epileptics), an effect of the tumour itself or a combination of the above. The pathophysiology of radiation-induced neurological damage is complex and imperfectly understood; it is thought to involve (1) an increase in permeability of the blood–brain barrier, (2) death of oligodendroglial precursor cells leading to demyelination, (3) subtle changes in neuronal activity and vascular damage leading to frank radiation necrosis and (4) loss of radio-sensitive stem cell compartments, which under the inflammatory stress, induced by radiation, preferentially undergo gliogenic maturation, as opposed to participating in neurogenesis (Mizumatsu *et al*, 2003; Soussain *et al*, 2009).

A recent review across a wide range of tumour types suggested that risk factors for radiation-induced neurological toxicity (NT) include both treatment variables (radiation dose, fraction size, conformality index, volume treated, overall treatment time, chemotherapy use) and patient variables (older age, diabetes mellitus) (Lawrence *et al*, 2010). We are not aware of any large studies that have specifically examined the NT of radiation treatment in subjects with high-grade gliomas, with a view to identifying risk factors and associations between acute and late toxicity, and eventual survival.

**Purpose**

By performing a retrospective analysis of RTOG high-grade glioma studies we sought to answer the following questions:

1. What is the incidence of acute and late NT following RT for high-grade glioma?
2. What are the risk factors for acute and late NT following RT for high-grade glioma?
3. Is there an association between acute and late NT?
4. What are the long-term implications of acute NT?

## Materials and methods

Patient data was pooled from 14 RTOG high-grade glioma trials that accrued a total of 2761 subjects (Table 1). Eligibility criteria were consistent in all of the studies: histologically confirmed supratentorial malignant glioma; age of at least 18 years; normal hepatic, renal and bone marrow function; and an interval of 6 weeks or less from surgery to initiation of radiotherapy. Ineligibility criteria included previous malignancies (except skin carcinomas), previous chemotherapy, or head and neck irradiation. All the trials combined RT with systemic anti-tumour therapy.

### Definition of acute and late neurological toxicity

‘Acute toxicity’ is defined as adverse events that occurred within 3 months of commencing therapy; events occurring after this were classified as ‘late’. RTOG Acute Morbidity Scoring Criteria and RTOG/EORTC Late Radiation Morbidity Scoring Schema were used for the following studies: 8302, 8409, 9006, 9305, 9411, 9417, 9513, 9602 and 9710. NCI – CTC version 2.0 and RTOG/EORTC Late Radiation Morbidity Scoring Schema were used for the following studies: 9803, 9806, 0013, 0021 and 0023. For the purposes of this report, we only considered NTs of grade 3 or greater, without regard to attribution. Owing to the database's design we were unable to scrutinise details of the NTs.

The trials analysed used a range of doses and fractionation schemes. The effects of different fractionation schemes on the normal brain were compared by calculating the biological-equivalent dose (BED) (Fowler, 1989) using a normal tissue alpha/beta ratio of 3 (Lee *et al*, 1998). The RTOG trial 9305 combined fractionated therapy (60 Gy, BED 100) with a single-fraction radiosurgical boost. Although there is no accepted way to convert this into a BED, we considered the BED to be ‘above 120’ for the purposes of statistical analysis.

### Statistical methods

Frequency distributions of patient survival time (survive ⩾3 month *vs* survive <3 months) for two groups (acute NT *vs* no acute NT) were compared using *χ*^2^-tests. McNemar's test was used to test the difference between two correlated proportions – occurrence/no occurrence of acute and late NTs. Logistic regression was used to assess the relationship between acute and late NTs. It was also used to assess the relationship between pre-treatment characteristics, treatment options and the occurrence of acute NTs. For the survival end point, the Kaplan–Meier method was used to estimate the rates, and the log-rank test was used to compare them between the two patient groups (acute NT *vs* no acute NT). The Cox proportional hazards (PH) model was used to estimate the hazard ratio (HR) associated with overall survival while adjusting patient-specific factors. A two-sided test was used at a significance level of 0.05 for all the evaluations.

Patients dying within 3 months of RT are by definition not able to develop late NT; they were therefore excluded from analyses of late toxicity.

## Results

A total of 2761 patients were accrued; 151 patients (5%) were excluded from the analysis because of being ineligible, no protocol treatment or withdrawal of consent leaving 2610 patients. Baseline characteristics are listed in Table 2.

Median follow-up of all subjects was 11.2 months; median follow-up of the 279 patients censored subjects who were still alive at last follow-up was 57.3 months (this difference in length of follow-up between all subjects (censored and uncensored) and censored subjects reflects the fact that the risk of dying apparently decreases after living up to a certain time). The 265 patients who lived less than 3 months were excluded from analyses of late toxicity. A total of 182 cases (7.0%, crude rate) of acute NT and 83 cases (3.5%, crude rate) of late NT were reported.

Pretreatment characteristics and treatment options were assessed in logistic regression models to predict the occurrence of acute CNS toxicities. Table 3 lists the results, based on the univariate logistic regression analyses. Histology, chemotherapy and BED were considered as non-statistically significant at the significance level of 0.1 and not included in the further multivariate logistic regression analysis. In a stepwise logistic regression model considering the remaining six variables, Zubrod performance status, previous surgery type, neurological function, mental status and twice-daily (BID) radiation were significantly associated with acute NT (Table 3).

Pretreatment characteristics, treatment-related variables and the occurrence of acute NT were assessed in logistic regression models to predict the occurrence of late CNS toxicities. The following variables were considered in the univariate logistic regression analysis: age at diagnosis (<50 *vs* ⩾50), surgery type (biopsy *vs* partial/total resection), neurological dysfunction (no dysfunction/minor *vs* moderate/severe), mental status (normal function *vs* minor/confusion), once-daily radiation (yes *vs* no), Zubrod performance status (0 *vs* 1/2/3), histology (GBM *vs* anaplastic astrocytoma), chemotherapy (yes *vs* no), BED (⩽120 *vs* >120) and acute CNS toxicities (yes *vs* no). Age, Zubrod performance status, type of surgery, neurological function, mental status and histology were considered non-statistically significant at a significance level of 0.1 and not included in further multivariate logistic regression analysis. In a stepwise logistic regression model considering the four remaining variables, once-daily radiation, BED and previous occurrence of acute NT were all statistically associated with late NT (Table 4).

The association between acute and late toxicity amongst subjects who survived at least 3 months was examined by means of McNemar's test. Among the 148 patients experiencing acute NT, 10 (7%) patients experienced late NT; among the 2197 patients not experiencing acute NT, only 73 (3%) patients experienced late NT, *P*<0.0001, suggesting that acute CNS toxicities are statistically associated with late CNS toxicities.

**Figure 1:**
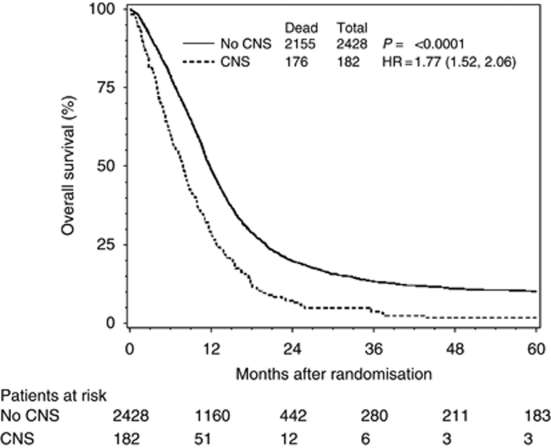
Overall survival, stratified by the presence/absence of acute neurological toxicity.

When the two patient groups (patients with and without acute NT) were compared with regard to overall survival, based on the log-rank test, a statistical difference was found (HR=1.77; 95% CI=1.52–2.06; *P*<0.0001). The median survival times were 7.8 and 11.8 months, respectively. The Kaplan–Meier curve is presented in Figure 1. Subjects with acute CNS toxicities were more likely to die within 3 months of treatment. Approximately 19% of patients with acute CNS toxicities died within 3 months, whereas 10% of patients without acute CNS toxicities died within 3 months, *P*<0.001.

**Figure 2:**
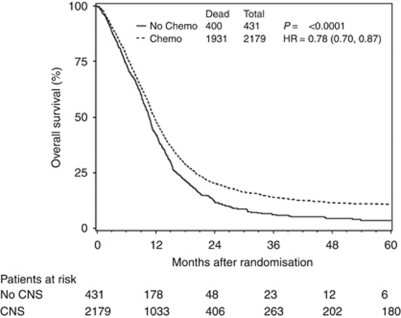
Overall survival stratified by the use of chemotherapy. Of note, 93% of subjects who received chemotherapy received BCNU. All patients who did not receive chemotherapy did receive a systemic agent (temozolomide, diaziquone, tamoxifen, thalidomide or β-interferon).

Recursive partitioning analysis (RPA) class (a combination of age, histology, Zubrod performance status, mental status, neurological function, symptom time and previous surgery) has been robustly established as a prognostic scale for patients with newly diagnosed high-grade glioma (Curran *et al*, 1993; Scott *et al*, 1998b; Mirimanoff *et al*, 2006). Recursive partitioning analysis class, BID radiation (yes *vs* no), chemotherapy (yes *vs* no), BED (⩽120 *vs* >120) and the occurrence of acute NT were assessed in a PH Cox model for overall survival (Table 5, Figure 2). Twice-daily radiation and BED were considered non-statistically significant and were not included in the multivariate Cox analysis. In a stepwise multivariate Cox model considering RPA class, chemotherapy and acute CNS toxicities, only RPA class and acute NT remained statistically associated with the overall survival (HR=1.43; 95% CI=1.2–1.7; *P*<0.0001) after adjusting for the RPA classes (Table 5).

## Discussion

We performed an analysis of the RTOG database to understand the risk factors and consequences of acute NT in patients with high-grade gliomas undergoing RT.

We found that both early and late toxicity are comparatively rare (3–7%) – in agreement with published experience (Dinapoli *et al*, 1993; Stupp *et al*, 2005; Keime-Guibert *et al*, 2007). Risk factors for acute NT that remained significant in the multivariate analysis were both patient (functional status, neurological function, mental status) and treatment (biopsy only, BID radiation) related. These findings, though novel in the field of brain tumours, are in keeping with the general oncology literature that frail patients experience more toxicity (Brian *et al*, 1995; Artz *et al*, 2006; Kumar Pal *et al*, 2010). The lack of association between chemotherapy and toxicity differs with the findings of the pivotal EORTC/NCIC phase III trial that established temozolomide and radiation as the standard of care. In that trial, in-field acute grade 3 and 4 toxicities (dermatological, infection and vision and nausea/vomiting) occurred in 7 and 14% of subjects in the control and temozolomide arms, respectively, (Stupp *et al*, 2005). This difference may reflect the type and extended duration of chemotherapy in the EORTC/NCIC trial. Conversely the rate of late toxicity reported by us (3.5%) is much higher than that reported in each arm of the EORTC/NCIC trial (<1%), it is not clear whether this is due to differences in treatment, population (the EORTC trial excluded older patients) or reporting practices. An important difference is that the statistics from the Stupp trial refer to any non-haematological toxic event, whereas the data presented here are specifically for NT.

Risk factors for late toxicity, significant in multivariate analysis, were once-daily radiation, high total radiation dose and previous acute NT. It is interesting to compare our findings with the recently published QUANTEC meta-analysis of the tolerance of the normal brain to irradiation, which investigated risk factors for late brain toxicity (Lawrence *et al*, 2010). Many of the studies analysed by the QUANTEC team involved the treatment of non-primary brain tumours (e.g., brain metastases and nasopharyngeal carcinoma). The QUANTEC authors demonstrated a sharp incidence in radiation necrosis when the BED rose above 120. Although the end points are not identical, in the current study we likewise found that a BED above 120, doubled the risk of late toxicity.

The association between acute and late toxicity has not previously been reported, and challenges the classic teaching that acute toxicity is fully reversible. A possible explanation is that these acute toxicities were so severe that healing was not possible; alternatively this may reflect a predisposition to toxicity amongst certain patients, possibly related to either tumour location (e.g., close to critical structures) or genetic makeup.

The relationship between acute NT and overall survival was unexpected. Patients who did not experience acute NT were found to have a 4 month longer median survival than those who experienced NT (of at least grade 3). This survival advantage was independent of RPA class. Although we lack a complete explanation, this may demonstrate the importance of normal tissue damage in determining long-term survival. A recent study likewise demonstrated that GBM patients who acquired motor or language deficits post-operatively had poorer overall survival than those who remained neurologically intact (Shinoda *et al*, 2001).

Our findings are in contrast with the association between pseudo-progression and improved prognosis in high-grade gliomas (Gerstner *et al*, 2009). Pseudoprogression is generally defined as radiological progression (oedema and sometimes contrast enhancement on MRI) soon after the completion of RT in patients with malignant gliomas, which is followed by spontaneous recovery and stabilisation (Brandsma *et al*, 2008). Pathologically it is thought to represent a mild form of radiation necrosis. Possible explanations for the difference between our findings and those associating pseudoprogression with a good prognosis are (1) pseudoprogression is especially associated with the use of temozolomide (Chamberlain *et al*, 2007; Brandsma *et al*, 2008). None of the patients in our study received this agent; rather the most frequently used systemic agent was BCNU, which appears to be much less potent. (2) Our patients were universally symptomatic, whereas most patients with pseudoprogression are asymptomatic. Hence, whereas pseudoprogression may be a form of intra-tumour necrosis, we suspect that the ‘acute toxicity’ cases described here represent damage to surrounding normal tissues. A more thorough understanding would require a case-by-case review of imaging, which unfortunately is not possible.

Despite the fact that our study dates from the pre-temozolomide era, we found that the use of chemotherapy was associated with increased survival (Figure 2). Of those who received chemotherapy, 93% received BCNU. As this association was only found on univariate, but not in multivariate analysis, its significance is unclear. Nevertheless, the association is in agreement with previous meta-analyses that have likewise identified methylating agents to be effective radiosensitsers in this disease (Chang *et al*, 1983; Fine *et al*, 1993; Spiegel *et al*, 2007).

A weakness of this retrospective study is our inability to assess completeness of reporting, and accurate attribution of neurological events. The definition of NT varied between the studies depending on the toxicity scale used; further we do not have descriptions of these events. A possible explanation of our findings associating acute NT with long-term survival is ‘misclassification bias’, that is, the treating physicians had difficulty distinguishing treatment-related side effects from tumour symptoms, and that what was reported as ‘acute toxicity’ was in fact early tumour progression. A close reading of our results however, supports the fact that these were indeed true treatment-related side effects: (1) We found that compared with once-daily radiation, BID radiation produced increased acute toxicity, but decreased late toxicity. This is entirely in keeping with classic radiobiology teaching of normal tissue damage. The lack of impact of fractionation scheme on overall survival (Table 5) further supports the supposition that this is related to normal tissue injury and not tumour control. (2) Conversely, histology (GBM *vs* anaplastic astrocytoma) had no impact on either acute or late toxicity, despite the more aggressive nature of GBM. If ‘NT’ was in fact a measure of ‘tumour progression’, a correlation would have been expected. (3) This ‘misclassification’ bias would be expected to especially affect subjects whose tumours progressed very early. Nevertheless, if patients who died within the first 3 months are excluded from the analysis, the survival benefit associated with lack of acute toxicity remains (HR=1.69; 95% CI=1.43–2.01; *P*<0.0001, Supplementary Figure).

A further weakness of our study is the small number of patients encountering toxicity. Although we analysed 2610 subjects, there were only 182 acute toxic events and 83 late toxic events. Confirmatory studies from other large databases, or population-based cohorts are therefore needed to validate our findings.

In conclusion we have elucidated the risk factors for NT amongst patients with high-grade glioma undergoing RT. These should be considered when designing eligibility criteria for clinical trials. The finding that acute NT predicts for both late NT and poor overall survival in patients not receiving temozolomide is provocative and requires validation. This phenomenon appears to be distinct to the ‘pseudoprogression’ seen when temozolomide is combined with RT.

## Figures and Tables

**Table 1:** Clinical trial data analysed

**Study**	**Phase**	**Study question**	**BID RT**	**Concurrent systemic agent**	**Number of analysable subjects**	**BED**	**Acute neurological toxicity**	**Late neurological toxicity**	**Overall toxicity**	**Survival**	**Ref.**
8302	I/II	Hyperfractionated RT, dose escalation	Y	BCNU	756	73.6–114.2	86	9	Acceptable	HC	(Curran *et al*, 1992)
8409	I/II	Role of AZQ	N	AZQ	54	74–95	11	0	NR	HC	(1998)
9006	III (R)	Conventional *vs* hyperfractionated RT	Y/N	BCNU	693	100–100.8	28	15	NR	HC	(Scott *et al*, 1998a)
9305	III (R)	Radiosurgical boost	N	BCNU	187	100, >120	6	4	Acceptable	HC	(Souhami *et al*, 2004)
9411	II	Dose escalation for small tumours	Y	BCNU	105	98.1–108	5	3	Acceptable	HC	(Coughlin *et al*, 2000)
9417	II	Role of tirapazamine	N	Tirapazamine	122	100	3	1	More toxicities at higher dose	HC	(Del Rowe *et al*, 2000)
9513	II	Role of topotecan	N	Topotecan	84	100	4	3	Significant haematological toxicity	HC	(Fisher *et al*, 2002)
9602	II	Role of paclitaxel	N	Paclitaxel	61	100	1	4	Acceptable	HC	(Langer *et al*, 2001)
9710	II	Role of beta-interferon	N	Beta-interferon	55	100	1	2	Acceptable	HC	(Colman *et al*, 2006)
9803	I/II	Conventional fractionation, dose escalation	N	BCNU	203	110–140	10	13	Acceptable	HC	(Tsien *et al*, 2009)
9806	II	Role of thalidomide	N	Thalidomide	125	100	19	12	Acceptable	Slightly better than HC	(Yung *et al*, 2001)
0013	II	Intra-tumoral bleomycin	N	Bleomycin	14	100	1	4	—a	—a	—a
0021	II	Role of tamoxifen	N	Tamoxifen	75	100	2	4	Acceptable	HC	(Robins *et al*, 2006)
0023	II	Role of stereotactic RT boost	N	BCNU	76	172	5	9	Acceptable	HC	(Cardinale *et al*, 2006)

Abbreviations: AZQ=diaziquone; BCNU=carmustine; BED=biological-equivalent dose; BID=twice daily radiation; HC=historical controls; N=no; NR=not reported; R=randomised; RT=radiation therapy; Y=yes.

a Small study, never published.

**Table 2:** Pretreatment characteristics, (*n*=2610)

	***N***	**%**
*Age*		
<50	907	34
⩽50	1691	65
Unknown/missing	12	<1
		
*Zubrod*		
0	1294	50
1, 2, 3	1304	50
Unknown/missing	12	<1
		
*Surgery*		
Biopsy	650	25
Partial/total resection	1900	73
Other/unknown/missing	70	2
		
*Neurological dysfunction*		
None/minor	1631	62
Moderate/severe	961	36
Unknown/missing	18	1
		
*Mental status*		
Normal function	1725	66
Minor, gross confusion	706	27
Unknown/missing	179	7
		
*RPA class*		
I, II	183	7
III	410	16
IV	1043	40
V, VI	844	33
Unknown	130	5
		
*Histology*		
GBM	2233	86
AA	250	10
Other	127	5
		
*Twice-daily RT*		
No	1397	54
Yes	1213	46
		
*Chemotherapy*		
No	431	17
Yes	2179	83
		
*Biological drug*		
No	2179	83
Yes	431	17
		
*Biological-equivalent dose*		
⩽120	2344	90
>120	266	10

Abbreviations: AA=anaplastic astrocytoma; GBM=glioblastoma; RPA=recursive partitioning analysis; RT=radiation therapy.

**Table 3:** Univariate and multivariate analyses/logistic regression for acute neurological toxicities (*n*=2610)

		**Univariate analysis**		**Multivariate analysis** b	
**Variable**	**Comparison**	**OR (95% CI)** a	***P*-value**	**OR (95% CI)** a	***P*-value**
Age	<50	—			
	⩾50	1.59 (1.1, 2.2)	0.008	—	—
Zubrod	0	—			
	1, 2, 3	2.72 (1.9, 3.8)	<0.0001	1.77 (1.1, 2.7)	0.010
Surgery	Partial/total resection	—		—	
	Biopsy	1.63 (1.2, 2.2)	0.0029	1.43 (1.0, 2.0)	0.038
Neurological dysfunction	None/minor	—		—	
	Moderate/severe	2.91 (2.1, 4.0)	<0.0001	1.80 (1.2, 2.7)	0.0054
Mental status	Normal function	—		—	
	Minor/gross confusion	2.48 (1.8, 3.4)	<0.0001	1.69 (1.2, 2.4)	0.0025
Twice-daily RT	No	—		—	
	Yes	1.79 (1.32, 2.43)	0.0002	1.67 (1.2, 2.3)	0.0025
Histology	AA	—			
	GBM	0.95 (0.6, 1.6)	0.84	—	—
Chemotherapy	No	—			
	Yes	0.79 (0.5, 1.2)	0.22	—	—
Biological-equivalent dose	⩽120	—			
	>120	0.84 (0.5, 1.4)	0.52	—	—

Abbreviations: AA=anaplastic astrocytoma; BED=biological-equivalent dose; CI=confidence interval; GBM=glioblastoma; OR=odds ratio; RT=radiation therapy.

a Odds ratio: the odds ratio of 1 indicates no difference between the two subgroups.

b Multivariate model derived from stepwise selection.

Variable(s) not included in final model: age (dropped out during the stepwise selection process).

Variable(s) dropped from modelling as not significant with reference variables: histology, chemotherapy, BED (not significant during the univariate setting).

**Table 4:** Univariate and multivariate analyses/logistic regression for late neurological toxicities

		**Univariate analysis**		**Multivariate analysis** b	
**Variable**	**Comparison**	**OR (95% CI)** a	***P*-value**	**OR (95% CI)** a	***P*-value**
Age	<50	—		—	—
	⩾50	0.95 (0.6, 1.5)	0.81	—	—
Zubrod	0	—		—	—
	1, 2, 3	0.93 (0.6, 1.4)	0.75	—	—
Surgery	Partial/total resection	—		—	—
	Biopsy	1.09 (0.7, 1.8)	0.73	—	—
Neurological dysfunction	None/minor	—		—	—
	Moderate/severe	0.76 (0.5, 1.2)	0.26	—	—
Mental status	Normal function	—		—	—
	Minor/gross confusion	0.86 (0.5, 1.5)	0.62	—	—
Twice-daily RT	No	—		—	—
	Yes	0.37 (0.2, 0.6)	0.0001	0.42 (0.2, 0.7)	0.002
Histology	AA	—		—	—
	GBM	1.76 (0.7, 4.4)	0.23	—	—
Chemotherapy	No	—		—	—
	Yes	0.64 (0.4, 1.1)	0.09	—	—
BED	⩽120	—		—	—
	>120	2.83 (1.7, 4.8)	<0.0001	1.98 (1.1, 3.4)	0.016
Acute CNS	No	—		—	—
	Yes	2.11 (1.1, 4.2)	0.03	2.40 (1.2, 4.8)	0.014

Abbreviations: AA=anaplastic astrocytoma; BED=biological-equivalent dose; CI=confidence interval; CNS=central nervous system; GBM=glioblastoma; OR=odds ratio; RT=radiation therapy.

a Odds ratio: The odds ratio of 1 indicates no difference between the two subgroups, less than 1 indicates a protective effect.

b Multivariate model derived from stepwise selection.

Variable(s) not included in final model: chemotherapy (dropped out during the stepwise selection process).

Variable(s) dropped from modelling as not significant with reference variables: age, Zubrod performance status, surgery, neurologic function, mental status, histology (not significant during the univariate setting).

**Table 5:** Univariate and multivariate analysis/cox proportional hazards model for overall survival

**Variable**	**Comparison**	**HR (95% CI)**	***P*-value**	**HR (95% CI)**	***P*-value**
RPA	I, II	—			
	III	3.24 (2.6, 4.1)	<0.0001	3.27 (2.6, 4.1)	<0.0001
	IV	5.41 (4.4, 6.7)	<0.0001	5.41 (4.4, 6.7)	<0.0001
	V, VI	10.10 (8.1, 12.6)	<0.0001	9.92 (7.9, 12.4)	<0.0001
Twice-daily RT	No	—			
	Yes	0.93 (0.9, 1.0)	0.09		
Chemotherapy	No	—			
	Yes	0.78 (0.7, 0.9)	<0.0001		
BED	⩽120	—			
	>120	0.99 (0.9, 1.1)	0.86		
Acute CNS	No	—			
	Yes	1.77 (1.5, 2.1)	<0.0001	1.43 (1.2, 1.7)	<0.0001

Abbreviations: BED=biological-equivalent dose; CI=confidence interval; CNS=central nervous system; HR=hazards ratio; RPA=recursive partitioning analysis; RT=radiation therapy.

Multivariate model derived from stepwise selection.

Variable(s) not included in final model: twice-daily RT and chemotherapy (dropped out during the stepwise selection process).

Variable dropped from modelling as not significant with reference variables: BED (not significant during the univariate setting).
