# Supplementary material for: Early toxicity predicts long-term survival in high-grade glioma
Source: Br J Cancer. 2011 Apr 12;104(9):1365–71. doi: 10.1038/bjc.2011.123 (PMC3101937; doi:10.1038/bjc.2011.123)

**Supplementary figure:** Overall Survival, stratified by presence / absence of acute neurologic toxicity, excluding those subjects who died within the first 3 months.


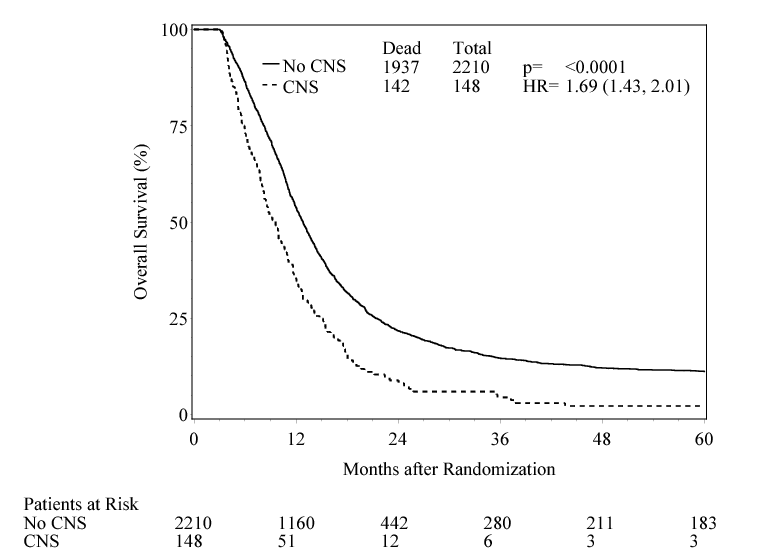

Supplement: Supplementary Figure 1 [file bjc2011123x1.doc]
